# Relative anterior spinal overgrowth in mild and moderate adolescent idiopathic scoliosis: a retrospective study

**DOI:** 10.1038/s41598-025-86912-0

**Published:** 2025-01-21

**Authors:** Haoyang Zhang, Xin Ye, Hongjiao Wu, Yi Shen, Yingsen Pan, Xiaoming Ying, Jiaying He

**Affiliations:** 1 Tuina department, Hangzhou Hospital of Traditional Chinese Medicine Hangzhou TCM Affiliated to Zhejiang Chinese Medicine University, Hangzhou, China; 2 The 3rd clinical medical college of Zhejiang Chinese Medical University, Hangzhou, China; 3 Tuina department, The 3rd affiliated hospital of Zhejiang Chinese Medical University, Hangzhou, China; 4 Tuina department, Hangzhou Hospital of Traditional Chinese Medicine Hangzhou TCM Affiliated to Zhejiang Chinese Medicine University, Hangzhou, China

**Keywords:** Paediatric research, Bone development

## Abstract

To determine whether relative anterior spinal overgrowth (RASO) occurs regardless of scoliosis segments and severity, and to explore the pattern of vertebral body height changes in adolescent idiopathic scoliosis (AIS). A total of 125 AIS and 179 non-scoliotic adolescents were enrolled. The anterior vertebral body height (VBHa) and posterior vertebral body height (VBHp) were measured on lateral spine radiographs, and the VBHa/VBHp ratio was calculated. The ratios were compared between the two groups and across scoliosis segments in the AIS group. The correlation between scoliosis severity and vertebral ratios, as well as the relationship between the apex vertebra’s ratio and Cobb angle, was analyzed. Results showed that the VBHa/VBHp ratios were higher in the AIS group than the control group from T6 to L5 (*P* < 0.001), with increasing ratios from T7 to T10 and L1 to L5. No significant differences were found across scoliosis segments. Pearson analysis showed positive correlations between scoliosis severity and ratios at T7, T8, and T11 (*P* < 0.05), and a negative correlation at L5 (*P* < 0.05). No correlation was found between the apex vertebra’s ratio and the Cobb angle. In conclusion, RASO is common in mild to moderate AIS and may help maintain spinal function.

## Introduction

Adolescent idiopathic scoliosis (AIS) is a three-dimensional spinal deformity involving the coronal, horizontal, and sagittal planes^1,2^. The importance of alterations of the sagittal plane in AIS patients has been increasingly emphasized in the diagnosis and treatment of scoliosis in recent years^3^. Sagittal spinal imbalance is also an important manifestation of AIS progression, as well as a predictor of poor prognosis^4^. Relative anterior spinal overgrowth (RASO), which is commonly proposed to explain the changes in the sagittal plane in AIS, has been suggested as a potential factor in the initiation and progression of AIS^5–7^. RASO was initially introduced as part of the etiopathogenetic mechanism of thoracic scoliosis. The changes in the anterior and posterior vertebral body heights of the thoracic vertebrae were favored in many previous studies of RASO, confirming the results of previous anatomical studies and supporting the consensus view that patients with thoracic AIS exhibit relatively faster growth of the anterior and slower growth of the posterior elements of the thoracic vertebrae, and that the disproportionate anteroposterior vertebral size is associated with the severity of the scoliotic curves^8–11^. According to Heuter-Volkmann’s law, asymmetric loading of the posterior parts of the vertebrae results from RASO, and leading to asymmetrical growth in all three planes of specific parts of the vertebrae. Asymmetrical growth of the neurocentral cartilage of the vertebra, for instance, has been shown to lead to AIS-like deformities in growing pigs, and could explain the development and progression of this deformity in humans^12^.

However, the presentation of RASO on the lumbar vertebral body in scoliosis has rarely been mentioned, and some researchers have questioned the theory of RASO in the pathogenesis of AIS^13–16^. In a recent study investigating vertebral morphology among patients with AIS, patients with Chiari I malformation (CMS)- associated scoliosis were compared with normal control subjects, with the results showing that the ratios for differential growth between the anterior and posterior elements of each thoracic vertebra in both the AIS and CMS groups were significantly larger than the ratios in the control group; however, there were no significant differences between the AIS and CMS groups for all mentioned parameters in the study. The authors thus concluded that RASO of the thoracic spine is not involved in the initiation of AIS, and the abnormal growth pattern of the vertebral body in AIS may occur as a secondary change to the spinal curve^15^. Another study by Schlosser et al.^16^ further demonstrated that the anterior-posterior height differences were much greater in the disks than in the vertebral bodies. The authors further concluded that this deformity was an adaptation of the forces acting on the spine rather than a primary disturbance of growth. Anterior overgrowth in AIS is not a generalized phenomenon, but is instead confined to the primary and compensatory curves, whereas the junctional zones do not present this growth discrepancy. As such, they speculated that anterior overgrowth was unlikely to drive the development of the deformity.

**Fig. 1:**
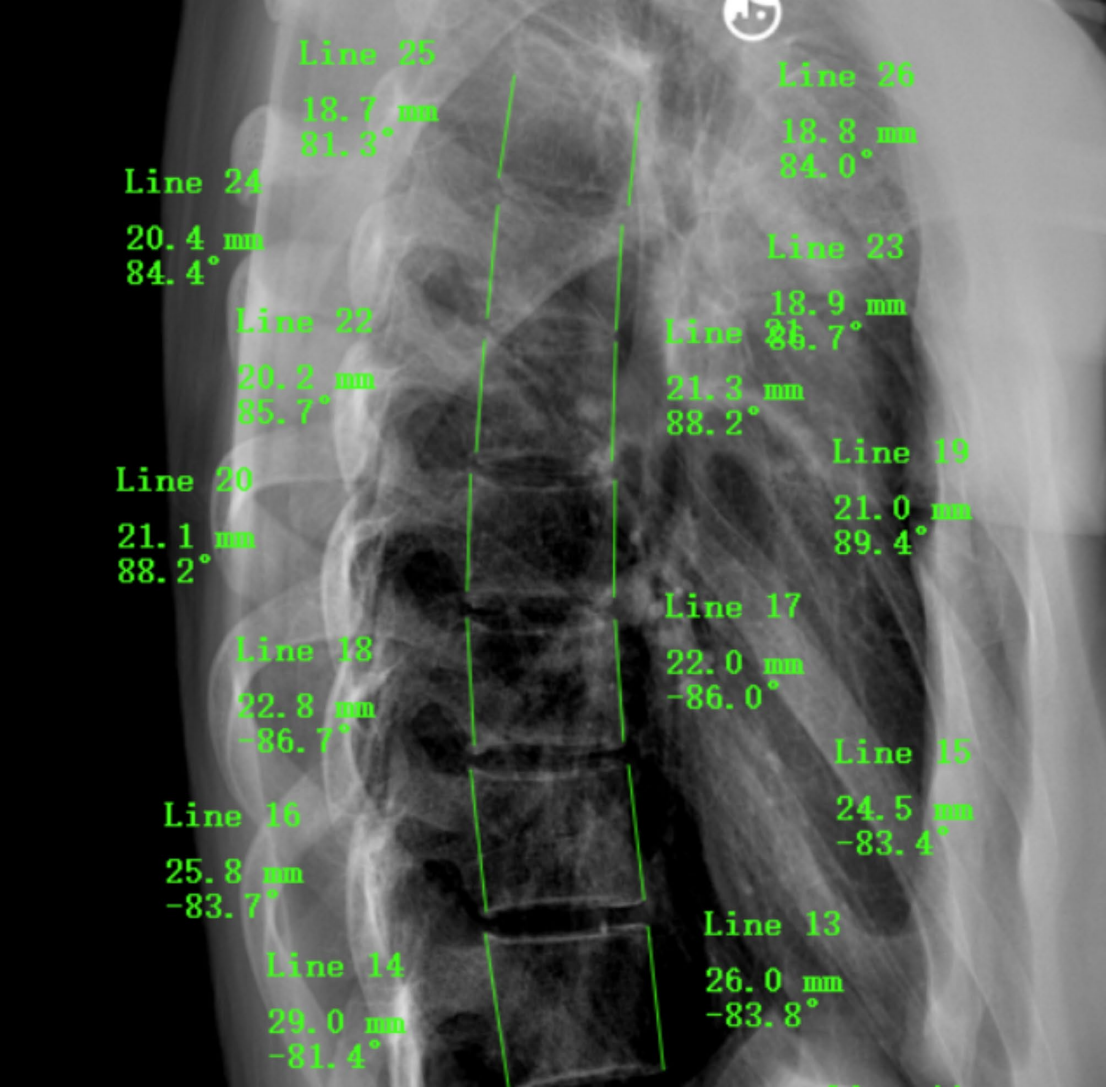
Diagram to show how measurements of the vertebral body on lateral spine x-ray (VBHa and VBHp) were obtained.

In the present study, we compared the ratios of the anterior vertebral body height (VBHa) to the posterior vertebral body height (VBHp) of the thoracic vertebrae (T6-T12) and lumbar vertebrae (L1-L5) in the AIS and control groups. The objective of this study was to observe whether the phenomenon of RASO is generalized regardless of scoliotic segments347-349 and scoliosis severity, and to clarify the regularity of vertebral body height changes in AIS, highlight the clinical importance of sagittal plane correction in the treatment of coronal plane scoliosis^17^, and provide a theoretical foundation for the early detection of spinal abnormalities during children’s growth (Fig. 1).

## Results

In total, 125 scoliosis patients with a mean age of 13.93 ± 2.21 years (range, 10–18 years) and a mean primary Cobb angle of 19.45° ±7.93° (range, 10.0°–41.7°) were recruited. There were 57, 35, and 33 cases with coronal apices on the thoracic, thoracolumbar, and lumbar spines, respectively. An additional 179 subjects with a mean age of 12.84 ± 2.10 years (range, 10–18 years) were recruited as the control group (Table 1).

**Table 1:** The numbers of vertebrae measured on different levels in two groups.

	T6	T7	T8	T9	T10	T11	T12	L1	L2	L3	L4	L5
AIS	68	109	125	125	125	125	125	125	125	125	125	125
Control	31	168	178	179	179	179	179	179	179	179	179	179

### Comparisons of ratios at the same segment of vertebrae between the two groups

**Fig. 2:**
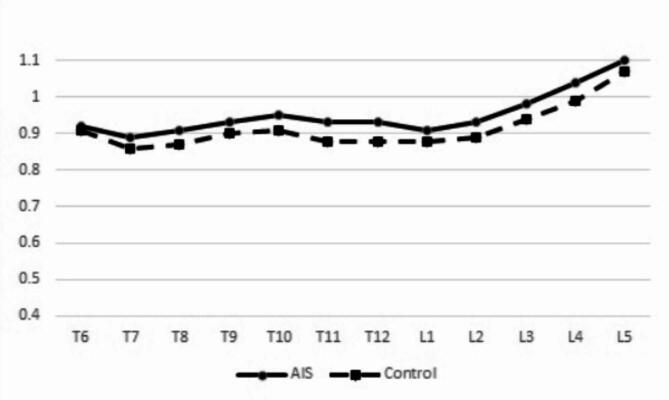
The mean ratios from T6 to L5 in AIS group and control group.

The ratios from T6 to T12 were higher in the AIS group than in the control group. Apart from T6 (*P* = 0.417), the differences between the two groups were statistically significant for all vertebrae (*P* < 0.001) (Table 2). The ratios of all lumbar vertebrae were also significantly higher in the AIS group than in the control group (*P* < 0.001for L1–L4, *P* = 0.007 for L5) (Table 3). Between T6 and L5, the VBHa to VBHp ratios in the AIS group were consistently higher than those in the control group (Fig. 2). The differences in the ratios over the same segments of different vertebral bodies between the two groups were similar, except for T6.

**Table 2:** Detailed ratios of the thoracic vertebrae (T6 to T12) in AIS and control groups.

	T6	T7	T8	T9	T10	T11	T12
AIS	0.92 ± 0.07	0.89 ± 0.08	0.91 ± 0.08	0.93 ± 0.07	0.95 ± 0.08	0.93 ± 0.07	0.93 ± 0.07
Control	0.91 ± 0.05	0.86 ± 0.06	0.87 ± 0.07	0.90 ± 0.07	0.91 ± 0.06	0.88 ± 0.06	0.88 ± 0.06
t	0.817	3.784	4.658	4.196	5.551	6.568	5.375
P	0.417	< 0.001	< 0.001	< 0.001	< 0.001	< 0.001	< 0.001

**Table 3:** Detailed ratios of the lumbar vertebrae (L1 to L5) in AIS and control groups.

	L1	L2	L3	L4	L5
AIS	0.91 ± 0.07	0.93 ± 0.07	0.98 ± 0.08	1.04 ± 0.09	1.10 ± 0.10
Control	0.88 ± 0.07	0.89 ± 0.07	0.94 ± 0.07	0.99 ± 0.08	1.07 ± 0.08
t	3.848	5.436	4.535	4.504	2.731
P	< 0.001	< 0.001	< 0.001	< 0.001	0.007

## Comparisons of the ratios of different vertebrae in the AIS and control groups

The ratios of the lumbar vertebrae were consistently higher than those of the thoracic vertebrae (Fig. 2). Ascending trends were observed in the ratios from T7 to T10 and from L1 to L5, but the ratios at T11 and T12 were both smaller than those at T10 in the two groups. The T10 ratios in the control and AIS groups were significantly higher than those in the T7, T8, T11, T12, T6, T7, T8, T9, and T12 groups (Table 4). Differences in the ratios of the different lumbar vertebrae were statistically significant in the AIS group (*P* = 0.043 for L1 and L2; *P* < 0.001 for the others). In the control group, the ratios of different lumbar vertebrae were also significantly different (*P* < 0.001), except between the L1 and L2(*P* = 0.199) (Table 5).

**Table 4:** P value of comparing each other of ratio on different thoracic vertebrae(T6-T12) in control group and AIS group.

	T7		T8		T9		T10		T11		T12	
	Control	AIS	Control	AIS	Control	AIS	Control	AIS	Control	AIS	Control	AIS
T6	+++	+	++	-	-	-	-	++	+	-	+	-
T7			-	-	+++	+++	+++	+++	++	+++	++	++
T8					+++	-	+++	+++	-	+	-	-
T9							-	+	+	-	+	-
T10									+++	-	+++	++
T11											-	-

**Table 5:** P value of comparing each other on different lumbar vertebrae(L1-L5) in control group and AIS group.

	L2		L3		L4		L5	
	control	AIS	control	AIS	control	AIS	control	AIS
L1	-	+	+++	+++	+++	+++	+++	+++
L2			+++	+++	+++	+++	+++	+++
L3					+++	+++	+++	+++
L4							+++	+++

Note for Tables 4 and 5: The symbols (plus sign) represent the sign of the difference, while the number of signs represents statistical significance (1: *p* < 0.05, 2: *p* < 0.01; 3: *p* < 0.001). minus sign represents nonsignificant differences.

## Comparisons of ratios among different segmental scoliosis in the AIS group

The greatest ratios of the thoracic and lumbar vertebrae were at T10 and L5, regardless of the coronal curve type. The smallest ratios of the thoracic vertebrae for thoracic scoliosis and lumbar scoliosis were at T7, but the smallest ratio for thoracolumbar scoliosis was at T8 or T12. The smallest ratios in the lumbar vertebrae for thoracolumbar and lumbar scoliosis were both on L1, but the smallest ratio for thoracic scoliosis was on L2. However, there were no statistically significant differences among the ratios for the different segmental scoliosis at any level (Tables 6 and 7).

**Table 6:** The differences of ratios on different segmental scoliosis for thoracic vertebrae.

	T6	T7	T8	T9	T10	T11	T12
Thoracic	0.91 ± 0.07	0.89 ± 0.08	0.92 ± 0.09	0.94 ± 0.07	0.95 ± 0.07	0.93 ± 0.07	0.93 ± 0.07
Thoracolumbar	0.93 ± 0.09	0.92 ± 0.07	0.91 ± 0.07	0.93 ± 0.08	0.96 ± 0.09	0.94 ± 0.08	0.91 ± 0.07
Lumbar	0.91 ± 0.07	0.88 ± 0.07	0.90 ± 0.05	0.93 ± 0.07	0.95 ± 0.08	0.93 ± 0.07	0.93 ± 0.08
F	0.464	1.852	0.935	0.240	0.428	0.346	0.823
P	0.631	0.162	0.396	0.787	0.653	0.708	0.441

**Table 7:** The differences of ratios on different segmental scoliosis for lumbar vertebrae.

	L1	L2	L3	L4	L5
Thoracic	0.93 ± 0.08	0.92 ± 0.07	0.96 ± 0.08	1.03 ± 0.09	1.09 ± 0.09
Thoracolumbar	0.89 ± 0.07	0.94 ± 0.07	0.98 ± 0.08	1.03 ± 0.08	1.10 ± 0.11
Lumbar	0.91 ± 0.07	0.94 ± 0.07	1.00 ± 0.06	1.05 ± 0.09	1.12 ± 0.10
F	2.022	1.096	1.935	0.935	0.770
P	0.137	0.337	0.149	0.395	0.465

## Correlation between scoliosis severity (Cobb angle) and VBHa: VBHp ratios in the AIS group

The Pearson coefficient for analyzing the correlation between scoliosis severity (Cobb angle) and the ratio of VBHa to VBHp showed a positive correlation between T6 and L3, and a negative correlation between L4 and L5. Statistically significant positive correlations were found for T7 (*P* < 0.05), T8 (*P* < 0.05), and T11 (*P* < 0.05), in addition to negative correlations for L5 (*P* < 0.05); however, these correlations were very weak (Table 8).

**Table 8:** Correlation between scoliosis severity (Cobb angle) and the ratios(A/P) in AIS group.

	T6	T7	T8	T9	T10	T11	T12	L1	L2	L3	L4	L5
Pearson coefficient	0.203	0.190*	0.195*	0.174	0.106	0.216*	0.115	0.065	0.103	0.017	-0.001	-0.186*

*Correlation is significant at the 0.05 level (2-tailed).

## The number of cases with the apex vertebra in AIS

As shown in the table, among the 125 AIS cases, the number of cases with the apex vertebra at T6, T7, T8, T9, T10, T11, and T12 were 0, 4, 10, 13, 9, 10, and 9, respectively. The number of cases with the apex vertebra at L1, L2, L3, L4, and L5 were 20, 29, 11, 0, and 0, respectively. (Table 9)

**Table 9:** The number of cases with the apex vertebra in AIS.

	T6	T7	T8	T9	T10	T11	T12	L1	L2	L3	L4	L5
AIS	0	4	10	13	9	10	19	29	20	11	0	0

## Correlation between the ratio of the apex vertebra of different segments of scoliosis and the Cobb angle

We investigated the correlation between the VBHa: VBHp ratio of the apex vertebra at different stages of scoliosis and the Cobb angle. The results showed that, regardless of the location of the apex vertebra, there was no significant correlation between the anteroposterior diameter ratio and the Cobb angle. (Table 10)

**Table 10:** Correlation between the ratio of the apex vertebra of different segments of scoliosis and the Cobb angle.

	T7	T8	T9	T10	T11	T12	L1	L2	L3
Pearson coefficient	-0.87	0.51	-0.02	-0.61	0.13	-0.11	-0.08	0.42	-0.56

*Correlation is significant at the 0.05 level (2-tailed).

## Discussion

The present study analyzed the ratios of VBHa to VBHp in the thoracic and lumbar spines of patients with AIS and non-scoliotic adolescents. The results showed that the ratios in patients with AIS were significantly higher than those in adolescents without scoliosis; in other words, the mean VBHa in patients with scoliosis was longer not only in the thoracic vertebrae, but also in the lumbar vertebrae. More than 100 years ago, Meyer^18^ and MacLennan^19^ stated that almost all cases of scoliosis were due to an inequality in growth between the posterior and anterior spinal columns. Roaf et al. subsequently reported that the basic lesion in scoliosis was the relative lengthening of the anterior components of the spine compared with the posterior elements^5^. Professor Cheng et al. later applied whole-spine magnetic resonance imaging (MRI) to re-investigate the height of both the anterior and posterior vertebral components in girls with AIS and in normal subjects. This study confirmed the results of previous anatomical studies, and supported the consensus view that patients with thoracic AIS exhibit relatively faster growth of the anterior and slower growth of the posterior elements of the thoracic vertebrae^8^. Vertebral torsion may also play an important role in the process of scoliosis resulting from RASO^20–22^.

In a study using three-dimensional MRI, Birchall D et al.^23^ previously supported the theory that torsion was a fundamental part of the early Patho mechanical process of AIS; they further hypothesized that there must be a consistent, long-term deforming force that results in a rotational torque being applied to the growing vertebral body, and it is likely that this force is fundamental to the pathogenesis of the scoliotic curve. Although the primacy of the sagittal plane in the pathogenesis of AIS has been questioned^13–16^, the obligatory involvement of the sagittal plane of the spine in curve initiation is generally accepted. numerous studies have shown that the sagittal plane in scoliosis patients differs significantly from that of normal adolescents, and changes in the sagittal plane play a crucial role in the prevention and treatment of scoliosis^24–26^. A study by Zhang C et al. pointed out that sagittal plane was a major risk factor for idiopathic scoliosis in adolescents. In AIS patients, lumbar lordosis is increased, cervical lordosis is reduced, and even cervical kyphosis occurs^17^. In recent years, research on vertebral body wedging in the sagittal plane supports the claim that scoliosis can be initiated through hypokyphosis;^27^ however, some studies have shown that there is no significant difference between sagittal wedge angles in mild and moderate scoliosis, and sagittal wedging has not been found to be an important component of the local vertebral deformity^15,16^. Therefore, we cannot support the theory that the precipitating factor for scoliosis initiation is anterior overgrowth or posterior tethering, which causes the spine to buckle^28^.

Moreover, only a limited number of studies have focused on the height of the lumbar vertebrae. In the present study, we found that the VBHa on the lumbar vertebrae grew faster, regardless of the scoliotic curve of the thoracic, thoracolumbar, or lumbar spine. The results showed that RASO arose simultaneously on the thoracic and lumbar vertebrae in AIS patients, regardless of where the scoliotic curve was, which is to say that the RASO was generalized not only on the vertebrae around the apex. However, Schlösser demonstrated that anterior overgrowth is not a generalized growth disturbance of the spine, but is instead confined to the area around the apex of both the primary and secondary curves^16^. This disparity in results is probably associated with the inclusion of patients with different scoliosis severities (mild and moderate scoliosis in our study and severe scoliosis in Schlösser’s study).

Although the ratios at the same level on the thoracic and lumbar vertebrae were significantly different between the AIS and non-scoliotic groups in the present study, consistent trends between the T6 and L5 were found for in both groups, with ascension from T7 to T10, descension from T10 to T12, and ascension again from L1 to L5. The differences in ratios at the same level on different vertebrae between patients with AIS and healthy adolescents were very similar; therefore, the two curves of the ratios were nearly parallel. The trend of alteration in ratios in our study was like that observed in a previous study^29^. As such, we speculated that RASO is not only a generalized condition in AIS, but that the velocities of overgrowth on different vertebrae are almost equivalent and coordinated with the growth of VBHp over the same segments. In other words, the anterior column of the vertebral body at the apex does not grow faster than the anterior column of the distal vertebrae. This phenomenon makes every vertebral wedging in the sagittal plane in patients with AIS consistent with the condition in healthy adolescents, and indicates that the spine is integrated to adapt to local deformities, such as scoliosis, to maintain spinal and truncal function in patients with AIS to the greatest extent possible.

Spinal sagittal malalignment in AIS with different segmental scoliosis has been reported in several prior studies. The results showed that thoracic kyphosis (TK) was significantly higher in the lumbar curves than in King I, King II, and King III curves. However, the difference in TK between the thoracolumbar curves and other groups was not significant. The degree of lumbar lordosis also tended to be higher for patients with a lumbar curve, although not significantly^30^. In another study, 49% of the curves presented sagittal malalignment in mild thoracic AIS, whereas only 13% of the (thoracal) lumbar curves and 6% of the no scoliosis adolescents were hypokyphosis^4^. Labrom et al. recently corroborated these findings in a longitudinal MRI study of AIS, finding that the changes in thoracic major coronal curve angle were positively correlated with increases in vertebral body wedging angles^31^. However, Mak et al. found that all AIS patients had a similar degree of thoracic kyphosis regardless of coronal curve type^32^. Another study also postulated that sagittal deformity is a generalized consequence seen across all scoliotic curvatures^33^. The results of the present study are largely in agreement with those of Mak et al. However, we found no significant differences in the ratios of thoracic, thoracolumbar, and lumbar scoliosis. The ratios were similar not only in the thoracic vertebrae, but also in the lumbar vertebrae despite the scoliotic site in the coronal plane. These results further confirm that RASO results from the overall effect on the spine rather than from the effect of local scoliotic levels in patients with AIS. This further confirms that sagittal plane correction is equally crucial during the scoliosis correction process.

Previous studies have reported different results regarding the correlation between RASO and scoliosis severity. A significant positive correlation between the scoliosis severity score and the ratio of the differential growth between the anterior and posterior columns for each thoracic vertebra was found in a prior study conducted by Guo^8^. In another study, the researchers also observed linear correlations between the coronal Cobb angle, axial rotation, and the anterior-posterior length difference (*r* = 0.729 for thoracic curves; *r* = 0.485 for (thoracal)lumbar curves)^16^. Vertebral wedging presented with mild scoliosis and increased as the scoliosis progressed^27^. However, De Smet et al.^34^ found no correlation between scoliotic segmental kyphosis and the degree of frontal Cobb curve angle. In another study, Newell et al.^35^ measured the changes in vertebral body height over time during scoliosis progression to assess how vertebral body height discrepancies change during growth, with the results showing that the degree of RASO was not related to the rate of progression or severity of the scoliotic curve, whereas AIS patients had a proportionally longer anterior column than non-scoliotic controls. The ratios of the different vertebrae did not correlate well with the coronal Cobb angles in this study. This observed significant correlations, which were very weak, were positive for T7(*r* = 0.190), T8(*r* = 0.195), and T11(*r* = 0.216), and negative for L5 (*r*= -0.186). In addition, our study also found that there is no significant correlation between the apex vertebra of the different segments of scoliosis and the Cobb angle. These results suggest that anterior overgrowth was unaffected by scoliosis severity in patients with mild and moderate AIS. We speculated that the differences in the results were probably due to the recruited subjects having different scoliosis severities. In our study, we excluded patients with severe scoliosis (Cobb angle > 45°)., whereas patients with mild, moderate, and severe degrees were included in Guo’s study, and most subjects had serious curves in Schlösser’s study.

Our study had some limitations. First, this was a retrospective single-center study. Second, the ratios between T1-T5 were not measured, and less data were available for T6 because these vertebrae could not be clearly observed on lateral full spine X-ray. In addition, no patients with serious scoliosis (Cobb angle > 45°) were recruited because of the limited number of patients with severe AIS in the study. In future studies, patients with mild, moderate, and severe scoliosis should be recruited so that changes in vertebral body height can be observed at different severities of scoliosis, and the correlation between RASO and scoliosis severity can be further researched.

## Conclusion

This study demonstrated that the ratios of VBHa to VBHp in the thoracic and lumbar vertebrae of patients with mild and moderate AIS were significantly greater than those in non-scoliotic children. The results confirmed that RASO in patients with mild and moderate AIS is a generalized phenomenon, regardless of the scoliosis severity and scoliotic segments. Therefore, sagittal plane correction plays an important role in the improvement of coronal plane scoliosis in patients with AIS. From these results, we speculate that the overgrowth on each vertebra is coordinated to ensure integration of the different vertebrae so that the vertebral wedging in the sagittal plane in patients with AIS is consistent with the condition in healthy adolescents, and that the spinal and truncal function in patients with AIS can be maintained as far as possible.

## Materials and methods

### Study participants

This was a retrospective observational study of the clinical data of AIS patients who underwent anteroposterior and lateral full-spine X-ray imaging at the 3rd Affiliated Hospital of Zhejiang Chinese Medical University between February 1, 2018 and June 30, 2020. The inclusion criteria were patients with a Cobb angle between 10° and 45°, aged 10 to 18 years, and diagnosed with a single-curve scoliosis with only one main curve, and no history of spine surgery. Patients were excluded if they had any associated non-idiopathic causes of spinal deformity. In addition, a control group comprising age-matched patients without scoliosis was enrolled. This study was performed in line with the principles of the Declaration of Helsinki. This study was approved by the Ethics Committee of the 3rd Affiliated Hospital of Zhejiang Chinese Medical University (ZCMU) (01/01/2018/No. ZSLL-KY-2017-045). Because secondary data were used, the need for informed consent was waived by the ethics committee of the 3rd Affiliated Hospital of ZCMU. The relevant guidelines and regulations performed all methods.

### Radiographic parameters

In the present study, several radiographic parameters were measured, including the coronal Cobb angle, VBHa, and VBHp. The Cobb angle was defined as the angle between the two most tilting vertebrae in the cranial and caudal regions^36^. For all subjects, VBHa and VBHp on lateral full spine X-ray were measured along these lines using Surgimap software (Nemaris Inc., New York, NY, USA) (Fig. 1). The classification based on the location of the main curve is as follows: thoracic scoliosis, with the apex vertebra located between the T1-T2 intervertebral disc and the T11-T12 intervertebral disc; thoracolumbar scoliosis, with the apex vertebra located between the T12 and L1 intervertebral discs; lumbar scoliosis, with the apex vertebra located below the L1-L2 intervertebral disc^37^. Each radiograph was measured by two observers, and an average height was calculated. The anteroposterior ratio was calculated as VBHa/VBHp. In some patients, VBHa and VBHp on the upper thoracic vertebrae, especially from T1 to T5, could not be measured on lateral x-ray, so VBHa and VBHp from T6 to T12 and all lumbar levels were measured; the numbers of measurements in both groups are shown in Table 1. The ratios at the same level between the two groups and among the different segmental scoliosis in the AIS group were compared. The correlation between scoliosis severity (Cobb angle) and the ratio of the different vertebrae was further analyzed.

### Statistical analysis

The mean and standard deviation (SD) were calculated for the ratio of each vertebra, and the differences in the mean values of each variable between the two groups were also analyzed. Two-group comparisons were performed using the student’s t-test according to the normality assumption. Similarly, more than two group comparisons were performed using ANOVA based on the normality assumption. Correlations between the Cobb angles and ratios were analyzed using Pearson’s correlation coefficient. Statistical significance for the type-I error rate was set at α = 0.05. SPSS software ver 18.0 was used for statistical analyses.

## Data Availability

The datasets used and/or analyzed during the current study are available from the corresponding author upon reasonable request.

## Abbreviations

AIS: Adolescent idiopathic scoliosis
RASO: Relative anterior spinal overgrowth
VBHa: Vertebral body height
VBHp: Posterior vertebral body height
